# Burden of caregivers of children with cerebral palsy: an intersectional analysis of gender, poverty, stigma, and public policy

**DOI:** 10.1186/s12889-020-08808-0

**Published:** 2020-05-08

**Authors:** K. Vadivelan, P. Sekar, S. Shri Sruthi, Vijayaprasad Gopichandran

**Affiliations:** 1 College of Physiotherapy, SRM Institute of Science and Technology, Kattankulathur, India; 2 Department of Paediatrics, SRM Medical College Hospital and Research Institute, SRM Institute of Science and Technology, Kattankulathur, India; 3 Department of Community Medicine, ESIC Medical College and PGIMSR, KK Nagar, Chennai, 600078 India

**Keywords:** Caregiver, Cerebral palsy, Psycho-social burden, Gender, Poverty, Stigma, Public policy

## Abstract

**Background:**

Caregivers of children with cerebral palsy suffer from a substantial psychosocial burden. However, there is a scarcity of documentation of the various sources of burden in low- and middle-income settings.

**Methods:**

We conducted qualitative in-depth interviews among mothers of children with cerebral palsy attending a physiotherapy facility. We purposively sampled mothers from rural and peri-urban areas in Tamil Nadu, India, till the point of data saturation. We analysed the transcripts using the socio-ecological model to identify the major dimensions of psychosocial burden among these mothers.

**Results:**

At the individual level the mothers perceived aches and pains due to the heavy physical activity of caregiving. They also suffered from a feeling of guilt about the child’s condition. Due to the difficulty in balancing family and work, they had significant financial burdens. They also perceived a lack of knowledge and awareness about possible options for the treatment of their child. At the interpersonal level, the mothers lacked support from their husband and family in the process of caregiving. They also had to suffer the ill effects of alcoholism and domestic violence from their husbands. They had to compromise on the care they provided to the other family members and their children without cerebral palsy. At the community level, the mothers had no support from the community members and felt isolated from others. The mothers also reported discrimination and lack of participation in social events. Environmental stressors like lack of inclusive public spaces, lack of options for public transport and unfriendly work timings and environment were major sources of burden. The mothers felt that the disability welfare support offered by the government was grossly insufficient and there was no platform for interactions with other peers and mothers suffering from a similar burden.

**Conclusion:**

Caregivers of children with cerebral palsy have unique burdens in a typical low- and middle-income setting including an intersection of gender norms, poverty, stigmatization and non-inclusive public policy, which need to be addressed to improve the quality of life of caregivers.

## Background

Cerebral palsy (CP) is one of the commonest causes of childhood disability in India. The condition is associated with neuromuscular spasticity, cognitive dysfunction, behavioural abnormalities, speech, visual problems and problems in feeding and gastrointestinal functions [1]. Therefore, children with CP are highly dependent on caregivers. Mothers are the most common caregivers of children with cerebral palsy. In low- and middle- income settings these mothers are often burdened with the care of the family, earning a livelihood as well as caring for the child with CP. This puts substantial burden and stress on the parents due to the intersection between gender norms, poverty, social stigma and caregiving for a child with a disability.

Caregiving has been shown impair the quality of life of the caregiver. Caregivers often suffer from stress and depression [2]. The level of stress and depression suffered by the caregiver is inversely proportional to their self-efficacy and level of social support [3].

The burden of caregiving for children with CP is a neglected phenomenon. In addition to the psychological problems described above, the parents also feel socially isolated, unable to participate in social life, stigmatized and develop conflicts in their family and society. Besides, they also undergo physical stress including lack of sleep, musculoskeletal aches and pains, and hypertension. Therefore, this neglected phenomenon of caregiver burden must be understood clearly and addressed [4].

A qualitative exploration of psychosocial stress among caregivers of children with CP done in India revealed that the main issues were disturbed social relationships of the caregiver, health problems, financial problems, worry about the future of the child and a need for more supportive services [5]. A similar Iranian study showed very similar stressors and also, rude and unsupportive interactions with the society [6].

The dimensions of caregiver stress in a typical south Indian population are not well documented. Therefore, this study was conducted as a qualitative exploration to understand the physical, psychosocial, financial, and other stressors among caregivers of children with CP in a typical low socio-economic south Indian context in Tamil Nadu. The specific objective was to identify the intersection between gender, poverty, social stigma and caring for a child with a disability in causing burden to the caregivers, as the intersection of these social determinants is likely to worsen the psycho-social stress of the caregivers.

## Methods

This study was conducted as a qualitative exploration with the methodological orientation of thematic content analysis. We conducted semi-structured in-depth interviews to obtain introspective, experiential, and personal data from the caregivers of children with CP. In-depth interviews are most suited to obtain personal and lived experiences of individuals along with their unique perspectives and opinions.

### Interviewers

The first and third authors conducted all the interviews, the former a male and latter a female. Both of us are practicing physiotherapists specialized in caring for children with CP. Most of the interviews were conducted by us together. As all the respondents were mothers of children with CP, we felt that having a female interviewer during the interviews will make the respondent more comfortable. Both of us had brief training in qualitative interview techniques. As we are long term care providers of the children with CP, we had a good rapport with the respondents before the interviews.

### Theoretical framework

The main theoretical approach of this exploration was the socio-ecological model of health. Bronfenbrenner’s socio-ecological model of health states that an individual’s health is influenced by multiple layers [7]. Individual-level influences, inter-personal relationships, social factors, organizational environment, and policy level determinants play a role. This model has been used to study behaviour change and the perceived health of people. We are applying this theoretical framework to understand the burden and stressors of caregivers of children with CP. The theoretical assumption is that the caregiver burden is influenced by the caregiver’s characteristics, interpersonal relationships, social interactions, social support and organizational structure and the policy environment.

### Sampling

Participants were mothers of children with CP. We sampled them purposively from among the mothers who brought their children for therapy to a tertiary care centre. We approached them face-to-face and obtained informed consent. There was no prior determination of a sample size. We conducted interviewsuntil the point of data saturation, which was obtained after conducting 9 interviews. The tenth interview was conducted to confirm the data saturation. All the participants who were approached consented to be interviewed. The respondents were from lower to lower-middle socioeconomic classes, living in rural and semi-urban areas around the tertiary care centre serving the community.

### Setting of interviews

We conducted interviews in the tertiary care centre in a private room. None other than the interviewers and the participants were present in the room during the interviews. After obtaining permission, the interviews were voice recorded using a digital voice recorder.

### Interviews

A total of 10 interviews were conducted. All the participants were women in the age range of 30 to 40 years, mothers of children with CP. The children were 5 boys and 5 girls between the ages of 2–10 years. A semi-structured interview checklist was used to facilitate the interviews. Extensive field notes were made by the interviewers during the interview. Each interview last for about 40 min to an hour.

### Transcription and analysis

We transcribed the data by carefully listening to the audio recording of the interviews. The transcription was carried out in the English language, translating the original interviews which we conducted in the local language Tamil. We performed a thematic content analysis. The first and last authors read the interview transcripts and performed open coding of the various statements. We then developed a coding tree. The first author used this coding tree to code the rest of the transcripts. The last author verified the codes and wherever there were disagreements, we resolved them by discussion and consensus. The coding was performed using a Microsoft Excel spreadsheet. After the first round of coding, we grouped the codes into meaningful themes. The broad themes were informed by the socio-ecological theoretical framework. We also identified verbatim quotes from the transcripts to support the various themes.

**Fig. 1:**
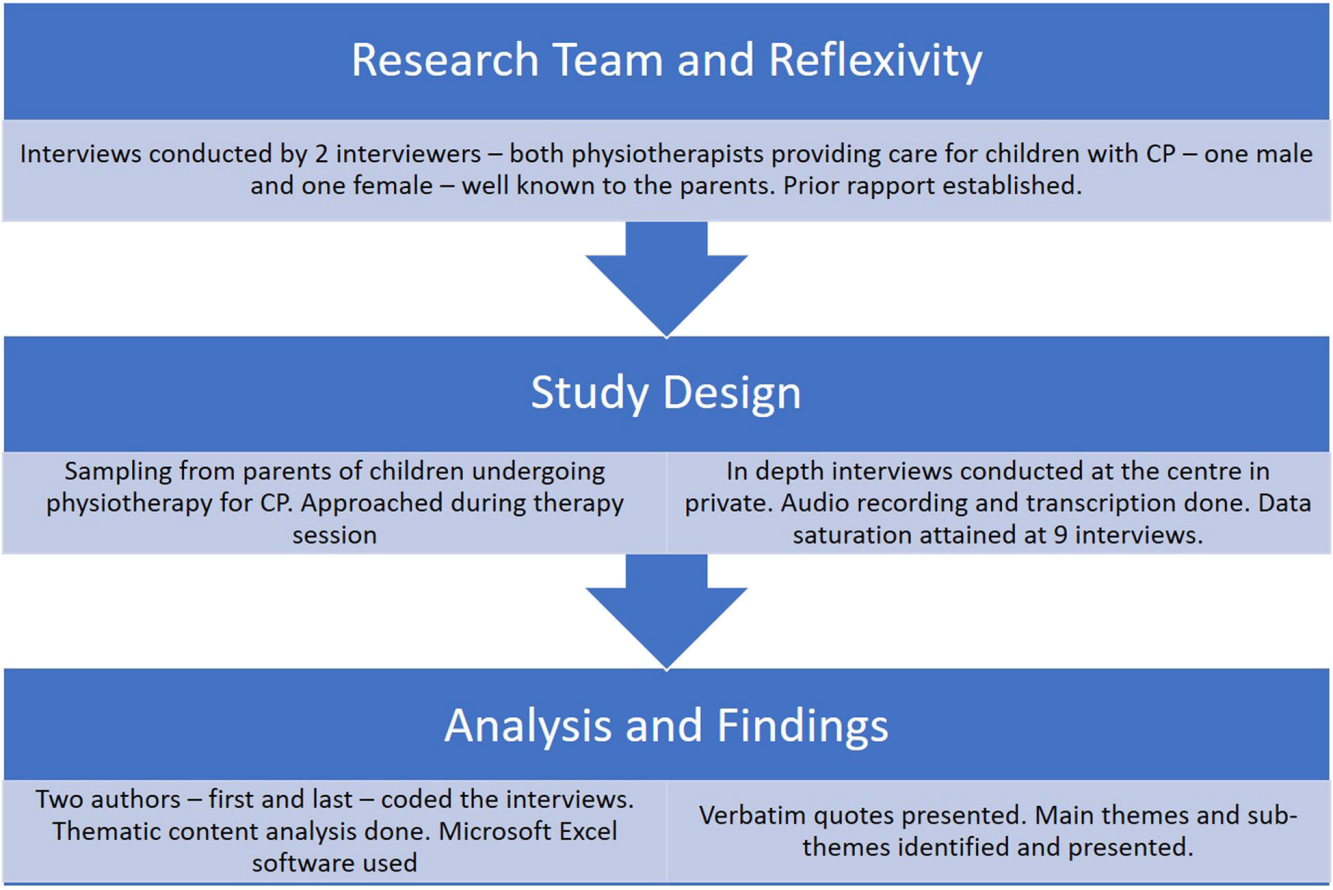
Study methods as per COREQ criteria

We attempted triangulation through coding by two researchers and verification and discussions with the other two researchers. The key elements of the methods are presented in the COREQ flowchart in Fig. 1.

The study was approved by the Institutional Ethics Committee (Human Studies) of Sri Ramaswamy Memorial Institute of Science and Technology, Kattankulathur with Approval No. 1505/IEC/2018, dated 25.10.2018. Written informed consent for interviews was obtained from all participants. The privacy and confidentiality of all the participants was strictly maintained.

## Results

Out of the total ten respondents of the in-depth interviews, all were mothers of 5 boys and 5 girls with cerebral palsy. The coding and analysis were based on the socio-ecological framework. We adopted the model to analyse the various levels of factors that act as stressors for caregivers of children with developmental disabilities. We present the results here under the various dimensions of the socio-ecological framework as shown in Table 1.

**Table 1:** Stressors of caregivers classified based on the socio-ecological framework

	Dimensions of the Socio-ecological framework				
	Individual stressors	Interpersonal stressors	Community stressors	Environmental stressors	Health system and policy stressors
**Physical**	The burden of work and lack of rest Physical aches and pains	Abusive husband, lack of physical support Inability to give good care for other children	No physical support from community members	Physical accessibility barriers Difficulty in public transport facilities	
**Emotional**	Guilt, blame, worry regarding the future of the child with a disability	Emotional abuse by spouse The guilt of not being able to care for other children	Sense of isolation in society	Worried about how to get around with the child	
**Social**	Blame by society on the parents	Blame by the society that she is not caring for the family	Discrimination in society	Non-inclusive spaces	Lack of social support groups
**Financial**	Unable to work, the financial burden borne by the individual			Lack of job options that help the caregivers have flexible hours	Disability welfare support insufficient
**Informational**	Lack of knowledge about caregiving options				Lack of support groups and information

### Individual stressors

There are several individual-level characteristics of the mothers who responded to the interviews which led to stress. These individual-level characteristics are typically their knowledge, awareness, physical abilities, beliefs, emotions and feelings. We found that the caregivers had many individual characteristics and attributes which precipitated stress.

#### Physical stressors

The primary caregiver of a child with a developmental disability is usually the mother and the major burden of household work is also usually thrust on her. Caregivers of children with developmental disabilities tend to bear a huge burden of physical work during the process of caregiving. This includes moving the child, cleaning the child, feeding him/her, providing physical therapy and playing with the child, etc. In addition to all this, she also has the work of the household and caring for other children and family members. The women do not have adequate time to rest and recuperate. The lack of rest led to aches and pains in the body and this hampered their ability to provide quality care for the child. This further led to stress.

“*I get some time for myself while my child is sleeping. And at that time also I sometimes prefer to complete all the remaining housework. Rest is not feasible in the present situation of my home*” – parent of a child with cerebral palsy

#### Emotional stressors

Mothers carried the emotions of guilt, blame and worry regarding the disability of their children. The feeling of guilt is strong in mothers. They felt that it must be something that they did during their pregnancy that led to this condition in their child. They also felt that giving birth to a child with a developmental disability is a failure of motherhood. The mothers also had to bear the blame that the society leveled on them.

“*The society still blames the parents for the child’s condition. Blame on the parents is very common.*” – parent of a child with cerebral palsy

Mothers worried about the future of their children. Their biggest concern was about the future of the child with a disability after their lifetime.

*“There is no future for my child. I am always worried about what will happen after our time.”* – parent of a child with cerebral palsy

#### Financial stressors

The burden of caregiving prevents mothers from participating in financially gainful jobs. Even the mothers who are working find it difficult to save money for the future. Invariably having a child with developmental disabilities leads to a financial burden. Many times, these financial burdens are borne by the caregiver alone. If a mother manages to save some money despite all these issues, that money gets spent on emergencies.

*“Planning for future saving is itself very difficult because we use any money saved for emergency purposes”* – parent of a child with cerebral palsy

#### Lack of knowledge

Though all the mothers who were interviewed expressed a high level of motivation and awareness about caregiving for a child with disabilities, lack of knowledge about options for caregiving, support systems, and other social welfare schemes was a major contributor to stress.

### Interpersonal stressors

Interpersonal relationships of the caregivers can act as buffers against stress. But sometimes they may also contribute to stress for the caregiver. Often caregivers report the lack of interpersonal support and help as the major stressors. Interpersonal relationships comprise of the immediate family including the spouse and other siblings of the child with a disability, extended family including parents, in-laws, and siblings of the caregivers, neighbors, and friends.

#### Lack of spousal support

The caregivers who were mothers of the children with disability perceived that their husbands were non-supportive. This was a cause for major stress for the caregivers. In addition to the lack of support, the mothers also felt stressed by their husband’s alcoholism. The mothers also feel emotionally stressed by the abuse at home. Often the abuse starts after the birth of the child with a disability.

“*He drinks smokes and comes home. I don’t allow my children near him when he is drunk. I lock them in a room where they will sleep at night. Even if my husband yells at me and beats me I will not open the door.*” – parent of a child with cerebral palsy.

#### Compromised care for other children

The caregivers reported having other children without cerebral palsy. They were stressed by the fact that they were unable to provide the same kind of attention and care for other children. They also felt stressed when they found others comparing the two children.

*“All say that I am wasting my time by caring for this child, and sometimes they say that I should take care of the second child (without cerebral palsy) properly.”* – parent of a child with cerebral palsy

“*I have two children, though I don’t compare the two children everyone else compares them and talk behind my back. This makes me feel hurt and spoils my mood.*” – parent of a child with cerebral palsy

#### Lack of support by extended family

Mothers who balance work, family as well as caregiving often feel stressed beyond their limits. They feel the need for support from their extended family. But they feel that extended families do not understand the troubles of caregivers and instead blame them.

“*Once in a while, we need to be dependent on others because managing family and kids is very difficult. But sometimes the family doesn’t understand about the child and speaks ill about me and my husband.*” – parent of a child with cerebral palsy

### Community stressors

Several characteristics of the community influence the stress in the caregiver. Community structures in urban and rural India are very different. In urban areas communities are individualistic with very little day to day interactions in neighborhoods. In rural areas communities are more open and day to day interactions are more. Based on this the extent of community stresses will vary. In this study, the majority of the participants are from rural areas. Therefore, the findings are representative of rural communities.

#### Social discrimination against the child and the family

Caregivers perceived a sense of discrimination and isolation from the community. They felt unable to participate in community events, celebrations or festivals. The caregivers were blamed for the condition of their child. They also felt that the community members spoke behind their backs about their child’s condition.

***“****I feel very low esteem by the way people around us look at us. I face a lot of problems while traveling by bus and interact with people.****”*–** parent of a child with cerebral palsy

#### No physical support from community members

Community members did not understand the special needs of children with CP. They did not adjust with such children and caregivers and openly blamed such parents for bringing their children in public.

*“In this society, there are two types of people some understand the child, and some do not understand the situation of the parents with children like ours. Sometimes the child will go and touch them, their saliva will drop on them. They get offended by these and do not understand.”* – parent of a child with cerebral palsy

### Environmental stressors

Several characteristics of the environment stress the caregivers. The environment has been identified as one of the major factors that influence the health and wellbeing of persons with disabilities [8]. Features of the environment, especially in low- and middle-income countries like India, pose a major barrier to the active engagement of persons with disabilities with society. The environment comprises the physical environment, the social environment and the attitudinal aspects of the environmental society. Some of the basic needs for a mother who cares for a child with a developmental disability are inclusive spaces where she can take the child out to interact with the society, ramps and footpaths where she can take the child out, disability-friendly public transport, good attitudes of the society towards children with disabilities and also workplaces which are understanding of the needs of a caregiver of children with special needs. When these are lacking, the caregiver is put through major stress.

#### Lack of accessible public transport

The main environmental interaction for many of the mothers caring for children with developmental disabilities in poor and low resource settings is travel. The caregivers mainly complained about the public transport services which were either completely inaccessible to a mother traveling with a child with special needs or had hostile co-passengers who made it difficult to travel.

*“Traveling on the public bus with my child is a problem. The buses are overcrowded. If I leave even one bus to take the next one, I will miss the physiotherapy appointment”* – parent of a child with cerebral palsy

*“When I travel with my child, often I do not get a seat. In the case of long journeys, I cannot eat in the middle of the journey and there are no proper restrooms for my child.”* – parent of a child with cerebral palsy

#### Lack of flexible timings in the workplace

Mothers who balance work, family responsibilities, and caregiving role, found it extremely difficult since the workplace timings are not flexible. Because of this many caregivers are unable to work and support the family financially.

### Health system and policy stressors

#### Insufficient disability support by the government

The government gives a disability pension of Rs. 1500 (USD 22) for every child with developmental disabilities. The caregivers felt that this amount is more of a token amount and is insufficient for providing good quality care for the child.

*“The disability card provides 1500 rupees every month, but it’s not enough for even the health care expenses.”* – parent of a child with cerebral palsy

#### Lack of buffers for health expenditures

Caregivers reported heavy health-related expenses, especially expenses related to traveling to a health facility for physiotherapy for the child. They felt that there were no buffers to meet these expenses. The social welfare policies of the government must cover at least some of these health care-related expenses.

*“I pawn my old jewelry for a sudden need for money as I face many financial issues related to my child’s care. I try to manage according to the situation at home and spend money. I do not want to compromise on the care for my child, whatever the financial situation maybe.”* – parent of a child with cerebral palsy

#### Lack of support groups and information support

The government does not organize any support groups of parents with disabilities or do not disseminate any information related to the welfare measures provided for such children. This is a major stressor as the caregivers do not know where to go and what help to seek.

## Discussion

To the best of our knowledge, this is the first qualitative study to explore in detail the dimensions of the stress of caregivers of children with developmental disabilities in the context of Tamil Nadu in south India. We identified important stressors in the socio-ecological framework including individual, family, community, environmental and policy domains [7]. Many of the stressors identified in this study are similar to ones identified elsewhere in India and the world [5]. Some of the findings such as guilt and self-blame of the parents, alcoholism of the husband and domestic violence experienced by the caregiver, non-inclusive public spaces and public transport and poor social welfare schemes for the families, are unique to this study and contribute to the broader understanding of stress and burden among caregivers of children with developmental disabilities in this context.

Individual-level factors such as physical discomfort and pain in various parts of the body of the caregivers due to the physical stress of caregiving have been described by other scholars [5, 9]. Similar physical aches and pains were identified in this study too. We noticed that the parents in our study perceived guilt and tend to blame themselves for the condition of their child. Previous systematic reports of such feelings of guilt and self-blame are not available in the literature. The parents tend to blame themselves for something they did wrong during the pregnancy for the condition of the child.

Another important individual level stressor was the lack of knowledge among the mothers about how best to care for their children. This has also been previously reported [5]. The lack of knowledge can lead to a feeling of helplessness and hopelessness. Addressing this is very important for improving the health and wellbeing of the caregiver. If the caregiver is provided adequate informational support, she can effectively care for the child. This, will alleviate the stress levels among the caregiver.

At the interpersonal level, we found that non-supportive family members were a major reason for worsening the stress. It made the mothers care for the child alone. This has been described in previous studies where family-centered caregiving is associated with a lesser amount of stress [10]. Lack of spousal support in caregiving has also been previously described as a major stressor [5, 11]. In our setting, alcoholism is a major problem among the men belonging to the lower socio-economic class. Alcoholism and associated domestic violence substantially increase the levels of stress.

Several studies have highlighted the importance of social support in caregiving [5, 12]. A community that does not understand the needs of a child with disabilities and a community that looks down upon such children greatly worsens the stress levels among caregivers. The mothers in our study felt discriminated against. They felt left out and isolated from society. This has also been described in previous studies [13].

One of the important highlights of our study was the emphasis on environmental factors which led to stress. The mothers we interviewed mentioned that non-inclusive public spaces and non-inclusive public transport are a major impediment for them to participate actively in this society. This is a great stressor for them in society. Not only does this restrict their movement and social participation, but it also places a huge financial burden on these caregivers.

Several contextual factors present in this study make the findings unique and important. The domains of stress identified in this sample of women relate to mothers who are the primary caregivers in most low- and middle-income settings [14]. Women, in general, have a level of vulnerability in these societies as a result of prevalent patriarchy in these societies. Most of the women who were interviewed were also from a rural background and lower socio-economic status. Thus, the intersection of gender, poverty, and rural background puts these women at a high level of vulnerability [15]. This high level of vulnerability is further compounded by the fact that they are burdened with the task of caregiving for their child with a developmental disability. This study highlights the important dimensions of stress in the presence of these contextual factors. This study identified that the important stressors in this context are gender-based violence exacerbated by rampant alcoholism, overburdening of women due to care-giving role because of gender norms, financial burdens complicating the stress due to poverty, and non-inclusive public spaces due to poor urban and rural planning.

The findings of this study have important implications for the practice of public health in the country. The fact that the caregivers of children with developmental disabilities most likely are highly vulnerable persons places a great responsibility on the public health system to provide support systems and take measures to ensure the wellbeing of these caregivers. Establishment of caregiver support groups can act as peer support systems where the caregivers can exchange notes, share information, provide physical, emotional and social support to one another. This study also provides important information that the financial support provided by the government as a welfare measure to children with disabilities is perceived to be insufficient. There is a need to revise the financial support. The government must take appropriate measures to improve urban and rural planning to make public spaces inclusive. Public transport must also have special provisions for caregivers traveling with children with special needs. The provision of these facilities will help reduce caregiver stress and burden.

There are several limitations to this study. The interviews with the caregivers were conducted in the health facility when they brought their children for physiotherapy. Therefore, the stressors that were identified are unique to the population who can afford and who are motivated enough to bring their children for therapy. These findings may not apply to those who cannot afford to bring their children for physiotherapy. A more purposive sampling of those women from the community might provide further information on stressors among them. Even among the women who were included and interviewed in the study, it was found that the focus of the interviews was health care for the child. This is probably because of the setting of the interviews and the researchers who conducted the interviews, who were therapists. Themes such as the caregiver’s relationship with the child, the caregiver’s relationships with their family and the rest of the members, and other dimensions of stress that bothers the caregiver never came up in the interviews. A more comprehensive study with purposive sampling in communities and interview by non-therapist researchers may help gain a deeper understanding. Despite these limitations, the study provides valuable information on the important stressors of caregivers of children with developmental disabilities.

## Conclusions

Caregivers of children with cerebral palsy have unique stressors and burdens in the south Indian context, which are dominated by the intersection of patriarchal gender norms, poverty, stigmatization, and poor public policy. A deeper exploration among a community-based sample of caregivers will help understand these stressors better for guiding public health policies to support the caregivers and improve their quality of life.

## Data Availability

The datasets used and/or analysed during the current study are available from the corresponding author on reasonable request. Please write to vijay.gopichandran@gmail.com

## Abbreviations

COREQ: Consolidated criteria for reporting qualitative research
CP: Cerebral Palsy
IEC: Institutional Ethics Committee
USD: United States Dollars
